# Accessory male investment can undermine the evolutionary stability of simultaneous hermaphroditism

**DOI:** 10.1098/rsbl.2009.0280

**Published:** 2009-07-15

**Authors:** Nico K. Michiels, Philip H. Crowley, Nils Anthes

**Affiliations:** 1 Animal Evolutionary Ecology, Institute for Evolution and Ecology, Eberhard Karls-Universität Tübingen, Auf der Morgenstelle 28, 72076 Tübingen, Germany; 2 Department of Biology and Center for Ecology, Evolution and Behavior, University of Kentucky, Lexington, KY 40506-0225, USA

**Keywords:** simultaneous hermaphroditism, sex allocation, sexual selection, male copulatory organ, adaptiveness, sexual antagonism

## Abstract

Sex allocation (SA) models are traditionally based on the implicit assumption that hermaphroditism must meet criteria that make it stable against transition to dioecy. This, however, puts serious constraints on the adaptive values that SA can attain. A transition to gonochorism may, however, be impossible in many systems and therefore realized SA in hermaphrodites may not be limited by conditions that guarantee stability against dioecy. We here relax these conditions and explore how sexual selection on male accessory investments (e.g. a penis) that offer a paternity benefit affects the evolutionary stable strategy SA in outcrossing, simultaneous hermaphrodites. Across much of the parameter space, our model predicts male allocations well above 50 per cent. These predictions can help to explain apparently ‘maladaptive’ hermaphrodite systems.

## 1. Introduction

Sex allocation (SA) theory links the evolution of simultaneous hermaphroditism and its stability against pure sex invaders to three prime conditions (Charnov *et al*. 1976; Charnov 1980; Fischer 1981). First, at least one sex function should show diminishing fitness returns, and thus fitness is maximized by combining optimal yields from both sexes in one individual. Second, diminishing returns are often associated with male function (Charnov 1979; Arnold 1994). The resulting less than 50 per cent male allocation provides more resources for egg production, so that population growth exceeds that of gonochorists with a 1 : 1 sex ratio. Third, conditions causing diminishing male returns are low density and mobility or poor mate searching (Tomlinson 1966; Ghiselin 1969), which reduce sperm competition and thus favour low male investment (Fischer 1984; Raimondi & Martin 1991; Schärer & Ladurner 2003). These models, however, are constrained in considering conditions for the evolution and stability of hermaphroditism, restricting the degree to which changes in SA in relation to group or body size are possible (Cadet *et al*. 2004; Brauer *et al*. 2007). Consequently, they may insufficiently predict evolutionarily stable SA when phylogenetically old, well-established hermaphroditic clades (possibly unable to change to gonochorism) are exposed to sexual selection. Moreover, few SA models consider male investments other than sperm, such as mate searching or seminal fluid compounds (but see Charnov 1996; Greeff & Michiels 1999; Pen & Weissing 1999; Crowley 2008).

Here, we explore how selection for male investments offering a disproportionate fertilization advantage affects optimal SA in outcrossing, hermaphroditic animals. In §5, we apply these to extant systems that apparently violate stability conditions for hermaphroditism.

## 2. Basic model

Let the fitness, *W*, of an outcrossing hermaphrodite be the sum of its fitness via female (eggs) and male function (offspring sired in *n* partners). Individuals mate on average *n* times as a donor and as a receiver. The environment determines mating opportunities, which are never rejected. Inseminations precede fertilization. Paternity per receiver depends on the investment, *m*, a donor makes per mating (e.g. ejaculate size) relative to that of the receiver's other mates. All remaining resources are allocated to eggs.

The *W* of a rare mutant introduced in a population with a SA denoted by overbars
2.1
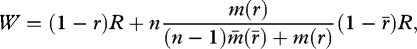

where *R* is the total resources for reproduction, *r* is the proportion of *R* allocated to male function (=SA), *m*(*r*) is the male investment per female mate as a function of *r* and *n* is the number of mates.

*m*(*r*) scales according to the number of matings
2.2
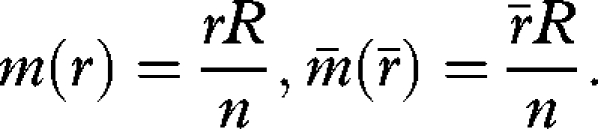



The evolutionary stable strategy (ESS) for *r* is obtained by setting the first derivative of equation (2.1) for *r* equal to zero, replacing 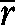 by *r* and solving for the ESS *r* = 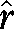


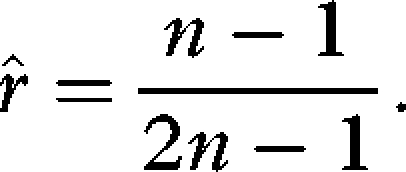

**Figure 1.**
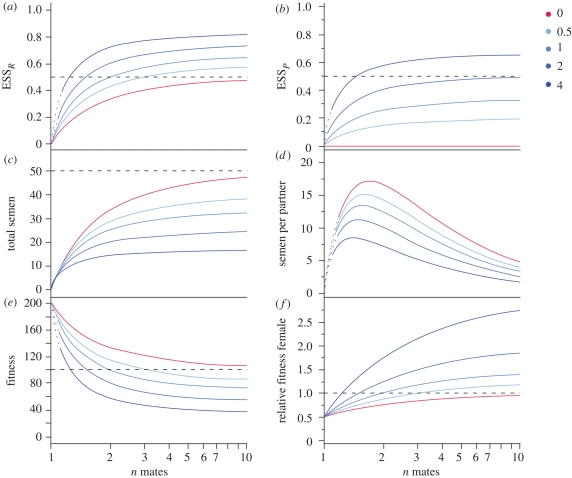
ESS solutions for five values of *a* as a function of mate number for (*a*) male allocation 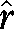 and (*b*) allocation to a male organ 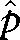. The other graphs were derived from these: total allocation to semen ((*c*) 
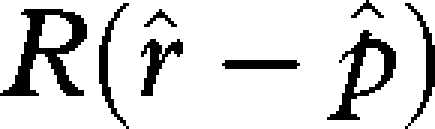
) and semen per partner ((*d*) 
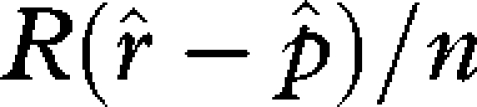
). Total fitness as sum of sired (male) and produced (female) (*e*) egg expenditure and (*f*) relative fitness of a pure female in a corresponding hermaphroditic population. *a* = 0 (red line) represents the traditional Charnov model.

This result is the same as that of Charnov (1980) and Fischer (1981) (figure 1, red line). It predicts 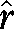 when sperm is the only form of male investment.

## 3. Accessory male investment in sperm competition

We now implement benefits offered by accessory male investment that can increase paternity but that trades off against sperm production *per se*. This investment could be structural (e.g. a penis, on which we focus in what follows) or functional (e.g. sperm plugs, manipulative seminal compounds, or behaviours that increase sperm competitiveness). By having a larger penis, own sperm may be put in a better position relative to rival sperm, offering a fertilization benefit in sperm competition or cryptic female choice (Eberhard 1985). The proportion of reproductive resources invested in this organ, *p*, is considered to be part of total male allocation *r*. Hence, *r* – *p* is the proportion of *R* left for sperm production. The fertilization advantage is modelled as a ‘virtual increase’ in sperm number by multiplying the resources invested per partner by *p*^*a*^. The dimensionless exponent *a* (0 ≤ *a*) allows us to control how *p* affects sperm precedence.

The investment strategy (2.2) is now additionally dependent on the benefit through a ‘male organ’ and changes into
3.1
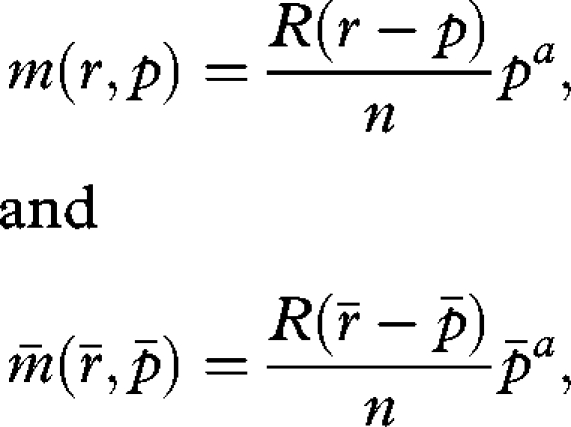

with *p* the cost of the male organ and *a* an exponent modifying the beneficial effect of *p* from nil (*a* = 0), decelerating (0 < *a* < 1), linear (*a* = 1) to accelerating (*a* > 1).

Substituting equation (3.1) into equation (2.1) and solving for the ESS values of *r* and *p* separately yields
3.2
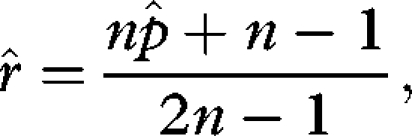

and
3.3
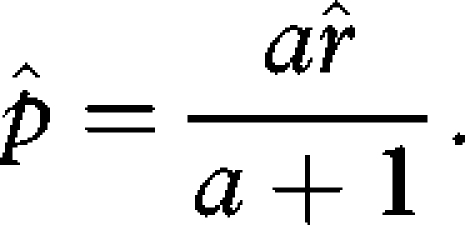



Now solving equations (3.2) and (3.3) simultaneously for 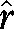 and 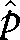 results in
3.4
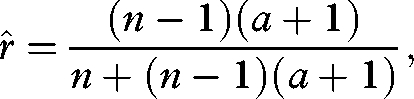

and
3.5
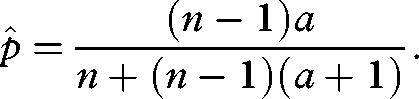

(Note: second derivatives of 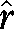 and 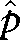 are negative, indicating maxima whenever *a* > 0 and *n* > 1.) The results show that even for few mates, adding a competitive advantage for a larger male copulatory organ (*a* > 0) drives 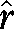 above 50 per cent (figure 1, blue lines). 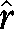 increases with *a*, rising to 80 per cent for accelerating effects. Note that for *a* = 0 (red line), 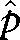 = 0, yielding an 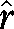 identical to the basic model above. The model further illustrates the trade-off between investments in sexually selected male traits (*p*) and investment in sperm only (compare 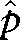 *R* with *total semen* in figure 1). All else equal, a decelerating advantage directs more male resources into sperm rather than accessory investment; the reverse is more likely for accelerating returns. The model also shows how ejaculate sizes are tailored depending on the number of mates (figure 1*d*). Under strict monogamy, 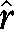 approaches zero but reaches a maximum with only *two* sperm donors competing for fertilizations, corresponding to theoretical predictions (Ball & Parker 1997; Wedell *et al*. 2002). With increasing numbers of partners, ejaculate sizes decrease owing to the requirement to divide the available amount of sperm across *n* partners.

## 4. Stability against pure sex invaders

Hermaphrodites are stable to invasion by females as long as their *per capita* egg production is ≥0.5 that of the average female (assuming a 1 : 1 sex ratio in the gonochorists and no paternal provisioning by males). Consequently, hermaphrodites with 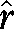 > 0.5 are prone to invasion by pure females. Under our paradigm, this situation is obtained across a wide range of the parameter space, indicating that such systems are inherently labile. Figure 1*e*,*f* displays the total fitness of a hermaphrodite, expressed as total amount of resources that contribute to an individual's female reproduction *plus* male fitness through the female partner (figure 1*e*) and the relative success of pure female invaders (figure 1*f*).

## 5. Discussion

We show that alternative investment options in addition to sperm generate selection regimes that diverge considerably from the original SA models, resulting in male allocations well beyond the 50 per cent threshold across a wide range of the parameter space. Such enhanced male allocation occurs even with a decelerating effect of the male structure *p* on sperm precedence (0 < *a* < 1). This enhancement strongly depresses population fitness and makes hermaphrodites inherently prone to invasion by pure females.

From a theoretical standpoint, pure hermaphroditic systems should thus be non-existent unless sperm competition is low. However, some extant hermaphroditic systems may be incapable of producing pure sex mutants. This appears particularly likely in clades where internal fertilization requires complicated structures for sperm transfer and fertilization. Cleanly switching off either of the two sex functions and making the resources fully available to the other function may be non-trivial, suppressing the success of pure sex individuals. Hence, unless developmental or genetic switches allow a simple breeding system reversal, hermaphrodites with high marginal benefit from accessory investment (high *a*) may be phylogenetic dead ends. As a second obstacle, behavioural systems such as conditional reciprocity may further lower the fitness of pure sex invaders, stabilizing hermaphroditism (Fischer 1981; Anthes *et al*. 2005). Unless replaced by a separate sex species that competes for the same set of resources, maladaptive hermaphrodites such as these may persist over long periods. An intermediate compromise may be facultative selfing, e.g. in pulmonate snails (Jarne & Auld 2006). This seems feasible whenever the cost of inbreeding is surpassed by the cost of selection for excessive male allocation and may explain the association of occasional aphally (the lack of a penis generating inability to donate and, in some species, inability to receive sperm) with selfing.

**Figure 2.**
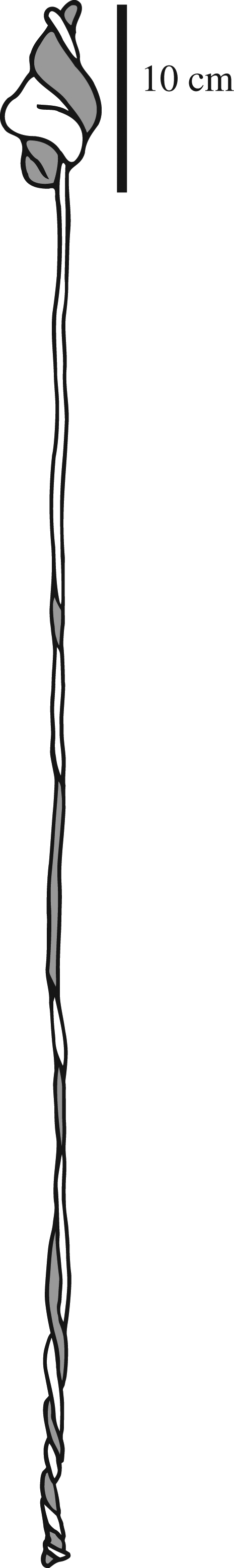
Schematic drawing of a mating pair of *L. corsicus*, illustrating the excessive size of the entwined male copulatory organs (drawn after a picture from M. Kaddatz and G. Falkner).

Spectacular, but also puzzling candidates for escalated male allocation exist among limacid land slugs. Pairs of *Limax corsicus* attach themselves to overhanging structures to evert and intertwine their gigantic male copulatory organs, hanging down for up to 92 cm in a 10 cm slug (Gerhardt 1933; figure 2). Sperm are exchanged at the penis tips. Large penises are apparently widespread among gastropods (e.g. Anthes & Michiels 2007; Reise 2007) and flatworms (N. K. Michiels 1992–1999, personal observation).

It is striking that transitions between hermaphroditism and gonochorism are frequent only in animals with external fertilization and correspondingly simple reproductive systems such as cnidarians, bivalves, crustaceans, polychaets and fishes (Harrison & Wallace 1990; Bauer 2004; Mank *et al*. 2006; Rouse & Pleijel 2006; Iyer & Roughgarden 2008). In contrast, clades with internal fertilization and advanced genital systems such as flatworms, oligochaetes, molluscs or insects show such transitions only rarely, if at all (e.g. Heller 1993); the reproductive evolution of these organisms may be constrained by phylogenetic inertia.

Future SA models should consider that the parameter space within which (some) hermaphrodites operate could be larger than previously assumed. They should be extended by including costs paid as a result of sexual conflict (Michiels & Koene 2006), mate searching (Puurtinen & Kaitala 2002) or selfing. All of these components may vary depending on the benefits of multiple mating, e.g. via fertility insurance or offspring performance (Sprenger *et al*. 2008). Finally, while we have alluded to possible systems that match our predictions, additional empirical measurements of SA and potential benefits of male accessory investments are clearly needed.
